# Leflunomide Induces Dose-Dependent Lung Injury in Mice via Stimulating Vimentin and NLRP3 Inflammasome Production

**DOI:** 10.3389/fphar.2021.631216

**Published:** 2021-04-23

**Authors:** Mohamed El-Sherbiny, Hoda Atef, Mohamed Ahmed Eladl, Abdelaty Shawky Mohamed, Mohamed El-Shafey, Howaida S. Ali, Sawsan A. Zaitone, Suliman Y. Alomar, Saeed Awad M. Alqahtani, Sheka Yagub Aloyouni, Mohammed A. Attia

**Affiliations:** ^1^ Department of Basic Medical Sciences, College of Medicine, AlMaarefa University, Riyadh, Saudi Arabia; ^2^ Department of Anatomy and Embryology, Faculty of Medicine, Mansoura University, Mansoura, Egypt; ^3^ Department of Histology and Cell Biology, Faculty of Medicine, Mansoura University, Mansoura, Egypt; ^4^ Department of Basic Medical Sciences, College of Medicine, University of Sharjah, Sharjah, United Arab Emirates; ^5^ Pathology Department, Faculty of Medicine, Mansoura University, Mansoura, Egypt; ^6^ Physiological Sciences Department, Fakeeh College for Medical Sciences, Jeddah, Saudi Arabia; ^7^ Department of Pharmacology, Faculty of Medicine, Assuit University, Assuit, Egypt; ^8^ Department of Pharmacology, Faculty of Medicine, University of Tabuk, Tabuk, Saudi Arabia; ^9^ Department of Pharmacology and Toxicology, Faculty of Pharmacy, Suez Canal University, Ismailia, Egypt; ^10^ Department of Pharmacology and Toxicology, Faculty of Pharmacy, University of Tabuk, Tabuk, Saudi Arabia; ^11^ Doping Research Chair, Department of Zoology, College of Science, King Saud University, Riyadh, Saudi Arabia; ^12^ Department of Physiology, Faculty of Medicine, Taibah University, Al-Madinah Al-Munawarah, Saudi Arabia; ^13^ Health Sciences Research Center, Princess Nourah Bint Abdulrahman University, Riyadh, Saudi Arabia; ^14^ Department of Clinical Pharmacology, Faculty of Medicine, Mansoura University, Mansoura, Egypt; ^15^ Department of Pharmacology, College of Medicine, AlMaarefa University, Riyadh, Saudi Arabia

**Keywords:** collagen 1, fibrosis, leflunomide, lung injury, mouse, NLRP3, vimentin

## Abstract

Recently, the therapeutic importance of the anti-rheumatic drug, leflunomide, has been increased after the involvement of leflunomide in treating other autoimmune diseases and its promising role in retarding human malignancies. Few studies have focused on the safety in human or animals without clear outlining of the pathologic features on target organs. One clinical study related leflunomide with significant pulmonary complications in predisposed individuals. The current study examined the dose-dependent lung injury produced by leflunomide in healthy mice. Albino mice were allocated into four different groups. Group (1): Vehicle control group, Group (2–4): mice received leflunomide (2.5, 5 or 10 mg/kg), respectively, for 8 weeks and then lungs were dissected from the mice for histopathological examination and fibrosis evaluation (Masson’s trichrome staining and α-smooth muscle actin immunohistochemistry). Enzyme linked immunosorbent assay was used to assess the vimentin and other inflammatory factors in the lung homogenate whereas Western blot analysis was employed to assess α-smooth muscle actin, vimentin and collagen 1. Results indicated that leflunomide induced dose-dependent pulmonary injury and the high dose and increased the vimentin, inflammatory markers (NLRP3 and interlukin-1β). Histologic examination showed distorted architecture, marked inflammatory cells infiltrate and increase collagen content. The findings were supported by Western blotting and the immunohistochemical study which showed greater pulmonary α-smooth muscle actin and vimentin content. In conclusion, the current results highlighted that leflunomide produced dose-dependent pulmonary toxicities that requires further investigation of the nature of injury.

## Introduction

Leflunomide has been permitted by the US Food and Drug Administration in 1998 for treating rheumatoid arthritis. Leflunomide exerts anti-inflammatory and immunosuppressive actions (Breedveld, 2000). It is used in active moderate-to-severe rheumatoid arthritis and psoriatic arthritis because it regulates the progressive nature of these diseases by inhibiting *de novo* biosynthesis of pyrimidine ribonucleotide. Leflunomide fulfills the desired goals of disease modifying antirheumatic drugs (DMARDs): it reduces the diseases signs and symptoms; it inhibits structural damage and improves the physical function (van Riel et al., 2004). The use of leflunomide is expanding during the last years but is accompanied by severe adverse reactions including hepatic, hematological, immune and respiratory systems (Toyokawa et al., 2007). Further, the long half-life of this drug is approximately two weeks; this delays resolving of some of the adverse reactions.

Leflunomide was reported to cause interstitial lung diseases (ILDs). Most of the affected patients’ presentation occurs within first three months of leflunomide regimen. Patients suffering from pre-existing ILDs are mainly at risk from this complication and avoidance of leflunomide in this population is considered as a must (Conway et al., 2016).

The innate immune system involves many pattern recognition receptors. There are five distinct classes of these receptors including the toll-like receptors (TLRs) and oligomerization domain (NOD)-like receptors (NLRs) (dos Santos et al., 2015). Activating the nod like receptor pyrins-3 (NLRP3) inflammasome needs a pair of pro-inflammatory stimuli. During the first stimulus, TLRs signaling stimulates nuclear factor-kB (NF-kB) and induces NLRP3 upregulation and pro-interlukin-1β (IL-1β). The second stimulus includes assembling of the multiprotein complex, followed by induction of self-cleavage of caspase-1. The previous reaction leads to pro-IL-1β cleavage producing the biologic active form (Wiese et al., 2017). Indeed, activation of NLRP3 inflammasome and consequent IL-1β maturation are associated in acute lung injury (ALI) leading to inflammatory reactions and/or fibrosis (dos Santos et al., 2015).

The cytoskeleton consists of microfilaments, microtubules and intermediate filaments (Fletcher and Mullins, 2010). Vimentin is an intermediate filament protein that is most abundant in the cells and possesses a crucial role in stabilization of its architecture (Eriksson et al., 2009). Vimentin was suggested to critically regulate NLRP3 (dos Santos et al., 2015). It was postulated that vimentin is essential for the assembly and NLRP3 inflammasome activation and leads to elevation in active IL-1β that participate in lung injuries in rodent models. Moreover, previous reports indicated that vimentin performs as an further checkpoint controlling the NLRP3 inflammasome (dos Santos et al., 2015).

The current study examined the dose-dependent lung injury induced by leflunomide and physio-pathogenetic mechanism underlying the lung damage. Hence, our study explored the effect of leflunomide on the pulmonary level of vimentin and NLRP3 as a possible mechanism may underlie the drug induced injury. As rheumatoid arthritis can affect the lung and results in interstitial pneumonitis (Lee et al., 2005), we have chosen in the current experiment to test leflunomide in healthy mice.

## Materials and Methods

### Experimental Animals

Forty male albino mice were housed in groups of five in hygienic conditions at the animal facility of the Faculty of Pharmacy in Suez Canal University after approval from the institutional research ethics committee (reference number 201603A7b). The mice were maintained normal dark/light cycle and temperature range 25–35°C. Food pellets and water were provided *ad libitum* throughout the study period.

### Design of the Experiment

The purchased mice were acclimatized for about 10°days and then allocated into 4 different groups, 10 mice in each of the experimental groups. Mice were gavaged with leflunomide (white powder from Sigma Pharmaceutical Company, Egypt) which was prepared in suspension form using 1% carboxymethylcellulose (CMC) solution (Bahr et al., 2015; Bilasy et al., 2015). Group (1): mice received CMC solution every other day, Group (2–4) mice received oral doses of leflunomide (2.5, 5 or 10 mg/kg) every other day, respectively. Doses were selected based on a previous study from our laboratory and were proven to induce organ toxicities in mice (Elshaer et al., 2019). 12°ml/kg volume of the drug (or vehicle) was administrated for a period of 8°weeks.

### Lung Dissection

After completion of this 8°weeks experiment, the animals were anesthetized with ketamine and sacrificed. A thoracotomy was performed to dissect the lungs; the right lobes were frozen at 80°C and the left lobes were perfused by fixation in a fixation solution (2% paraformaldehyde). The perfused lungs were excised and processed for light microscopic study.

### Homogenization of Frozen Lungs and Enzyme Linked Emmunosorbent Assays

Frozen lungs were weighed and homogenized as 10% in phosphate-buffered saline (PBS) using a Teflon homogenizer. The homogenates were centrifuged to remove solid materials and obtain the clear lysates which were quickly frozen for subsequent use in different assays. Samples from the lung lysates were analyzed by sandwich enzyme immunoassay kits for vimentin (MBS450175), NLRP3 (MBS7606270), IL-1β (MBS726898) from MyBiosource. The readings were taken by an ELISA reader (Stat Fax 2100, United States).

### Light Microscopy and Image Capturing

The formalin fixed lung lobes were washed, dehydrated and embedded in heated paraffin wax. After this step, 5°μm lung specimens were stained with hematoxylin and eosin (H&E) for demonstration of lung injury caused by leflunomide. Lung injury was assessed semiquantatively according to Ashcroft histological index, scoring index which grades from 0–7 (Ashcroft et al., 1988) and Masson’s trichrome stain for demonstration of fibrosis. In the stained sections, collagen fibers appear blue, nuclei appear black, cytoplasm, muscle, erythrocytes appear red (Bancroft and Stevens, 2001).

Lung injury was assessed semiquantatively according to Ashcroft histological index, scoring index which grades from 0–8 (Grade 0): Normal lung (grade 1): minimal alveolar or bronchiolar wall fibrous thickening of (grade 3): moderate alveolar or bronchiolar wall thickening with no noticeable damage of lung architecture (grade 5): greater fibrosis with definite loss of the architecture and development of fibrous bands or small masses of fibrous tissues (grade 7): honeycomb lung and (grade 8): total fibrous obliteration of the lung tissue.

In every microscopic field, the complete zone of the microscope image was outlined and the investigator first took the decision whether the parenchyma was fibrotic or normal. In case of normal tissue predominates in the zone, it was assigned a zero score whereas in case that fibrotic tissues predominates the investigator took the decision on the level of fibrosis that predominates in the zone. The investigator tried to assign each field by one of the odd numbered grades. If the investigator faced difficulty in taking a decision for the degree of scoring, the zone was given the intervening even score (2, 4 or 6) between two odd numbers (Ashcroft et al., 1988).

### Immunohistochemistry for Alpha-Smooth Muscle Actin and Vimentin

About 3–4 µm cut sections from the lung blocks were fixed to positive charge adhesive microscope glass slides. Sections were subjected to deparaffinization and then were treated with 0.2% H_2_O_2_ prepared in phosphate-buffered saline for 30 min for blocking endogenous peroxidases. Next, the sections were incubated in a humid chamber with primary antibodies overnight. Antibodies were: rabbit polyclonal anti-vimentin antibody, dilution 1:100 (Cat# GTX100034, GeneTex, USAUnited States) and rabbit anti-α-smooth muscle actin (α-SMA antibody, 1:50, Cat# A19607, ABclonal, MA, United States). The sections were rinsed three times in phosphate-buffered saline and then incubated with secondary antibody (goat anti-rabbit peroxidase-conjugated streptavidin) for 1 h at room temperature. The washing step was repeated again three times. Visualization of immunoreactivity was done by adding 3,3′-diaminobenzidine as a chromogen then counterstaining was performed using Mayer’s hematoxylin. In another section, the primary antibody was replaced by phosphate buffer solution, while other steps of the procedure were the same; this served as a negative control section.

### Image Analysis

The % of area covered by the blue color in Masson’s trichrome staining or immunostaining area for vimentin and α-SMA were estimated by ImageJ (Ali et al., 2019). The areas were determined in six equally spaced fields in each lung section in a blinded manner. The slides were inspected by an Olympus CX51 microscope and images were taken using an Olympus digital camera (E-620, United States).

### Western Blot Analysis of α-Smooth Muscle Actin, Vimentin and collagen1

Lung tissues were homogenized in RIPA buffer (Santa Cruz Biotechnology, CA, United States). Total protein from the frozen lung tissues was extracted using the BCA protein extraction kit and processed according to a common method described previously (Ali et al., 2015; Suliman et al., 2018). 50°µg proteins were denaturized and then loaded in polyacrylamide gels and separated by electrophoresis. Samples were subsequently transferred to a polyvinylidene difluoride membrane (Amersham Biosciences, Bucks, United Kingdom). After that, the membrane was blocked by in 5% nonfat dry milk in 1% Tris-buffered saline with Tween 20 (TBST-20) and 3% bovine serum albumin for 2 h. The polyvinylidene difluoride membranes and primary mouse specific antibodies of SMA (catalog # 14–9760–82), vimentin (catalog #MA3-745), collagen-1 (catalog # PA5-95137), and β-actin (catalog #PA1-183) were obtained from ThermoFisher Scientific (Waltham, MA, United States) incubated for 16 h at 4 C and washed four times with TBST-20. Then, proteins were incubated with HRP-conjugated secondary antibodies for 2 h at the room temperatire. TBST80 was used to wash the blot and the reaction was visualized using Super Signal West Femto substrate (ThermoFisher Scientific). Film bands were captured and quantified for intensities by normalization to the β-actin bands. The quantification process was achieved by ImageJ software (NIH, United States) (El-Ghaiesh et al., 2020).

### Statistical Analysis

Quantitative data were tabulated and checked for the goodness of fit by K-S test. Data showed goof fitness were presented as mean ± standard deviation of the mean (SDM). However, the ordinal data related to Ashcroft scoring for lung sections were presented in box-plots and analyzed by Kruskal-Wallis ANOVA and Dunn’s test. All comparisons were set at *p* value <0.05.

## Results

**FIGURE 1:**
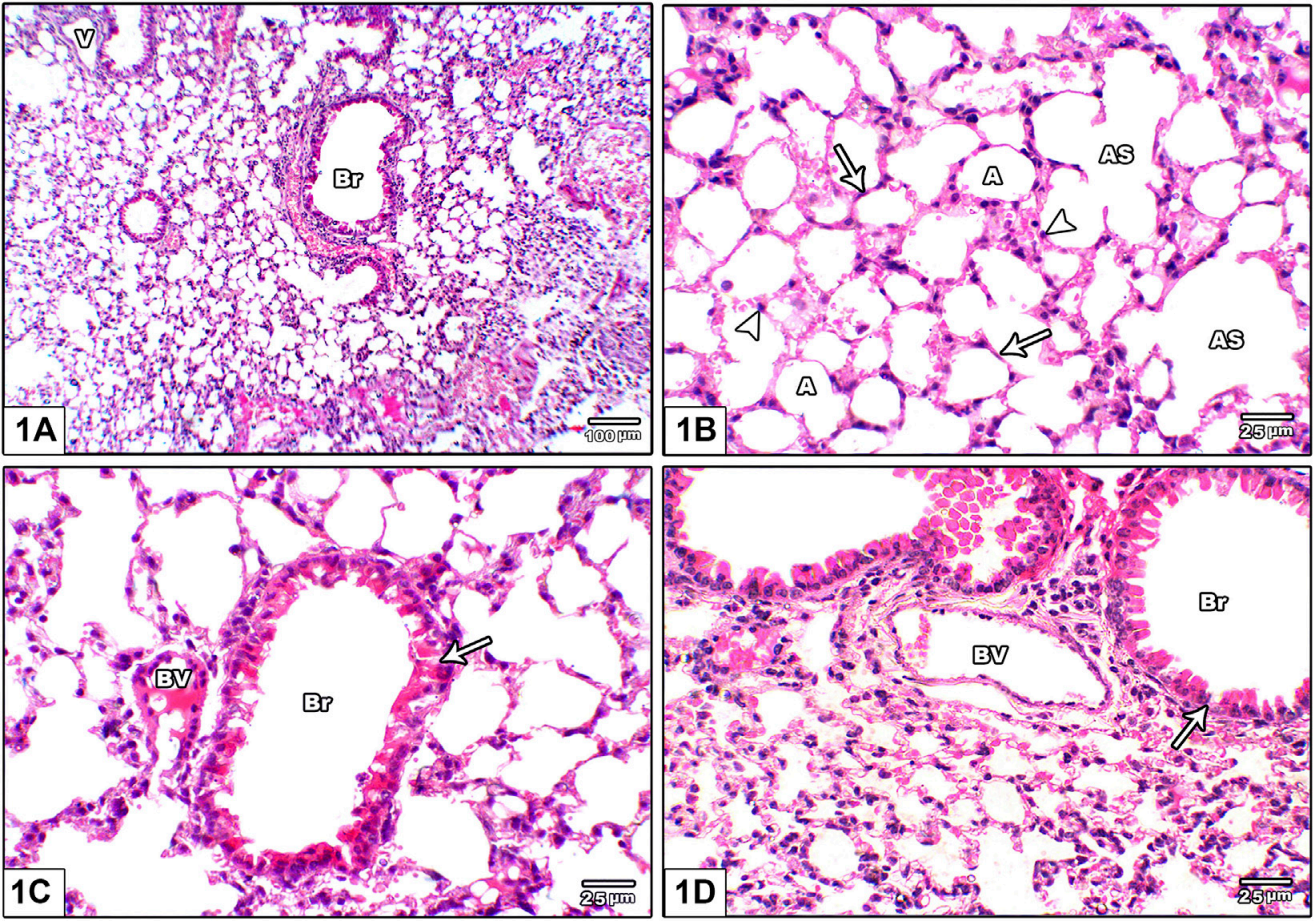
Photomicrograph of H&E stained lung from the vehicle control group **(A)** An image shows normal lung architecture with thin alveolar septa, normal bronchioles (Br) and normal blood vessels (V) **(B)** An image shows patent alveoli **(A)** with thin walls open into alveolar sacs (AS). The alveoli are lined by flattened pneumocyte type I cells with flat nuclei (arrow) and dome shaped pneumocyte type II with central rounded nuclei (head arrow) **(C)** and **(D)**: Images show bronchioles (Br) lined by intact folded respiratory epithelium with thin spiral layer of smooth muscle around it (arrow), note the juxtaposition of pulmonary blood vessels to bronchioles (BV).

Histopathological assessment of lung injury based on routine H&E staining demonstrated that the vehicle group showed normal lung architecture with thin alveolar septa, normal bronchioles and normal blood vessels. The alveoli are lined by flattened pneumocyte type I cells with flat nuclei. Bronchioles were lined by intact folded respiratory epithelium with thin spiral layer of smooth muscle around it (Figure 1).

**FIGURE 2:**
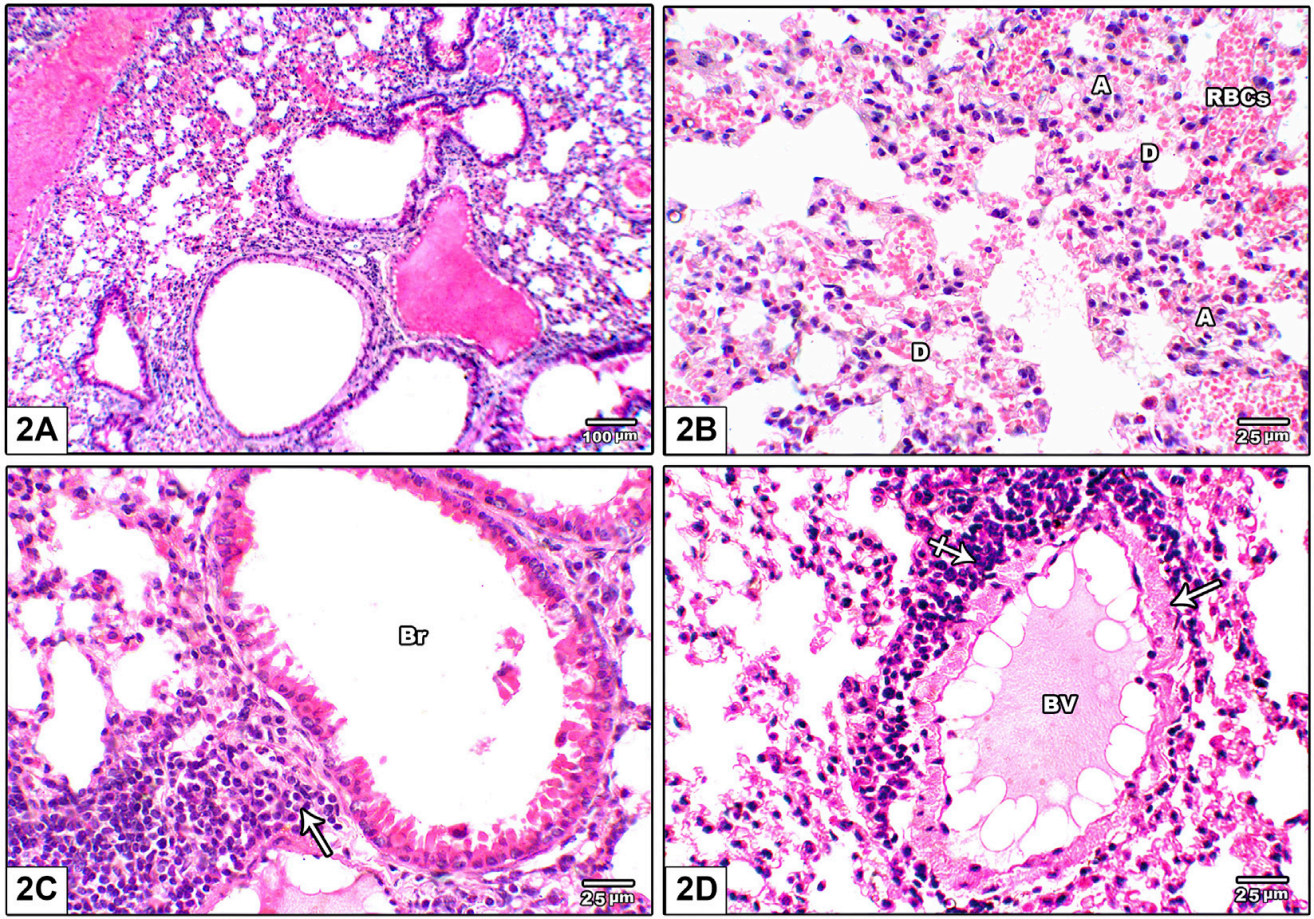
Photomicrograph of H&E stained lung from leflunomide 2.5 mg/kg group **(A)** Altered lung architecture with mild infiltration by mononuclear inflammatory cells, zones of non-regular air spaces with damaged inter-alveolar septa and narrowing alveoli **(B)** An image shows presence of collapsed alveoli **(A)** and some dilated ones **(D)** with thickened interalveolar septa and mononuclear inflammatory cell infiltrate in pulmonary interstitium with mild interstitial and alveolar hemorrhage (RBCs) **(C)** An image shows widening of the interstitium by mononuclear inflammatory cell infiltrate (arrow) **(D)** An image shows congested pulmonary blood vessels (BV) with minor perivascular cuffing by inflammatory infiltrate (crossed arrow) and increased wall thickness (arrow).

Figure 2 shows photomicrographs of H&E stained lung from leflunomide 2.5 mg/kg group. Panel (A) shows altered lung architecture with mild infiltration by mononuclear cells, zones of unequal air spaces and damaged inter-alveolar septa and appearance of narrowing alveoli. Panel (B) shows presence of collapsed alveoli and some other dilated alveoli with thickened interalveolar septa and mild interstitial and alveolar hemorrhage. Panel (C) shows widening of the interstitium by mononuclear inflammatory cell infiltrate while Figure 2D shows congested pulmonary blood vessels with minor perivascular cuffing by inflammatory infiltrate (crossed arrow) and increased wall thickness.

**FIGURE 3:**
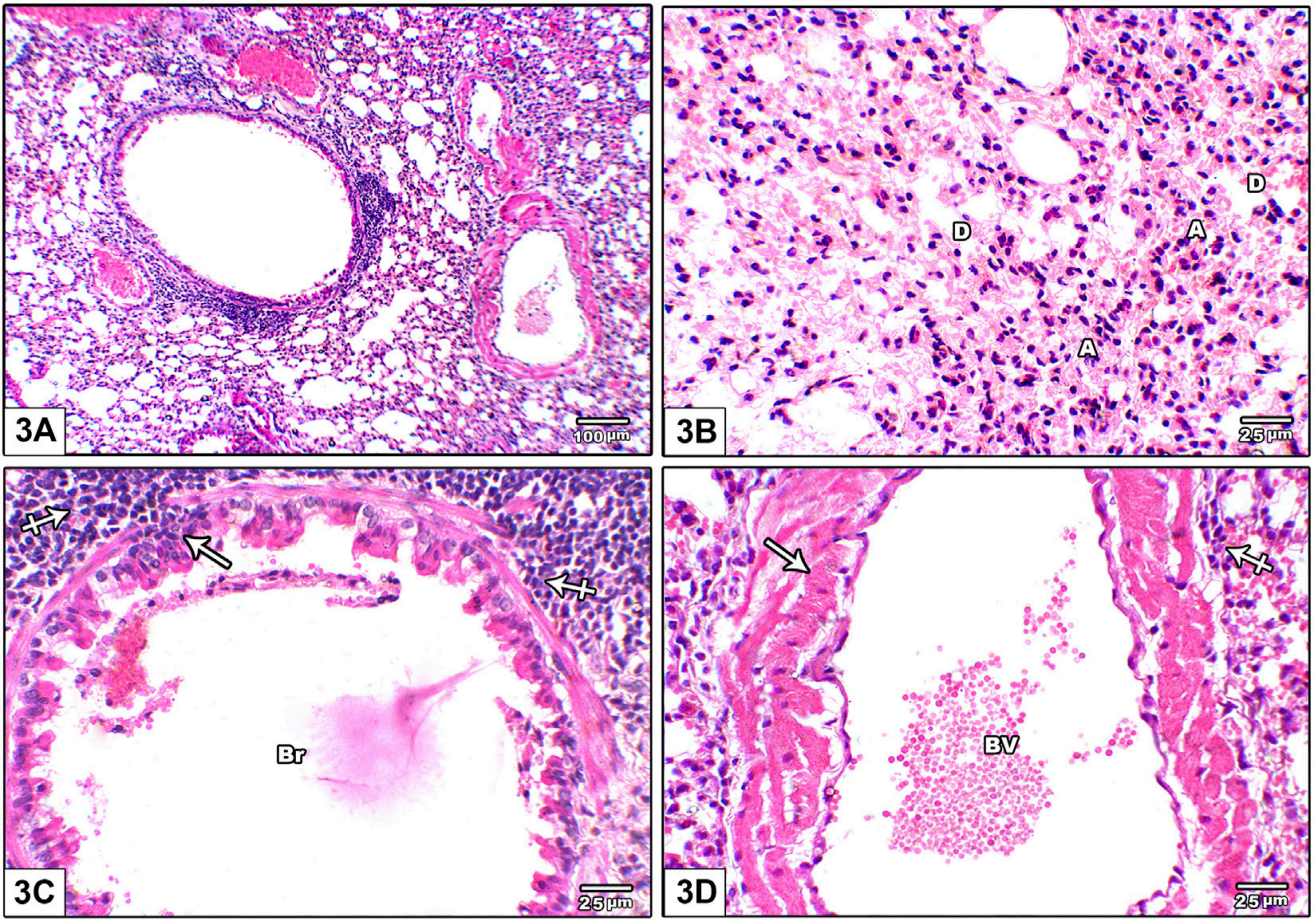
Photomicrograph of H&E stained lung from leflunomide 5 mg/kg group **(A)** An image shows disorganized lung architecture with mononuclear inflammatory cell infiltrate **(B)** An image shows areas of diffuse alveolar damage, pulmonary interstitium inflammatory cells infiltrate with interstitial alveolar hemorrhage. Most alveoli are collapsed **(A)** and few are dilated **(D) (C)** An image shows bronchiolar lumen (Br) with cellular debris (arrow) is seen with marked inflammatory cell aggregate around it (crossed arrow). Smooth muscle around it was thickened and heavily infiltrated with inflammatory cell (crossed arrow) **(D)** An image shows congested pulmonary blood vessels (BV) showing increased wall thickness (arrow) with foci of inflammatory infiltrate around it (crossed arrow).

Figure 3 shows photomicrographs of H&E stained lung from leflunomide 5 mg/kg group. Panel **(A)** shows disorganized lung architecture with mononuclear inflammatory cell infiltrate. Panel **(B)** shows areas of diffuse alveolar damage, pulmonary interstitium inflammatory cells infiltrate with interstitial alveolar hemorrhage. Panel **(C)** shows bronchiolar lumen with cellular debris with marked surrounding inflammatory cell aggregates. Smooth muscle around it was thickened and heavily infiltrated with inflammatory cell. Panel **(D)** shows congested pulmonary blood vessels showing increased wall thickness with foci of inflammatory infiltrate around it.

**FIGURE 4:**
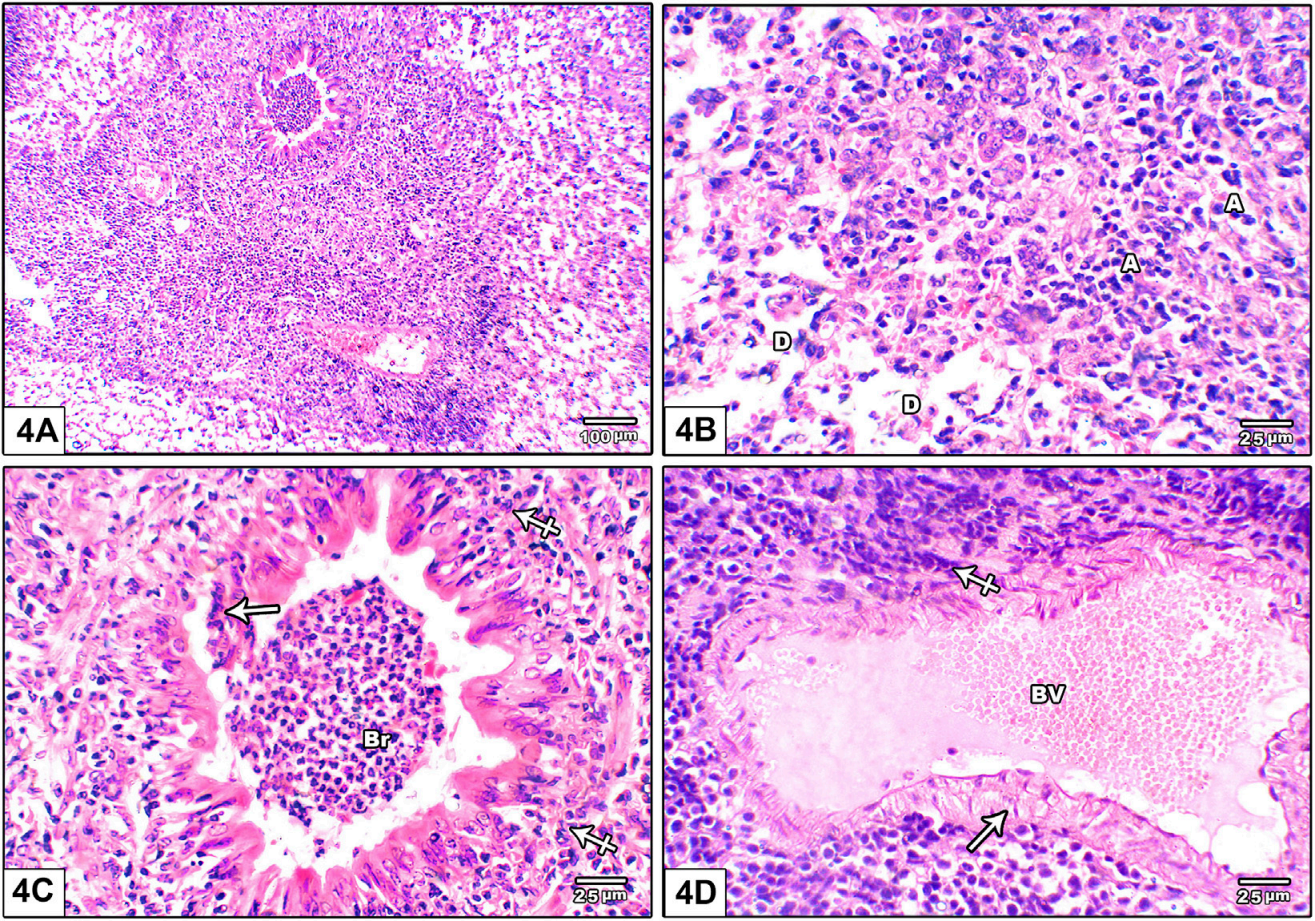
Photomicrograph of H&E stained lung from leflunomide 10 mg/kg group **(A)** An image shows markedly distorted lung architecture with heavy mononuclear inflammatory cell infiltrate **(B)** An image shows multiple areas of diffuse alveolar damage, thickened pulmonary interstitium with inflammatory cells and interstitial alveolar hemorrhage. Increased collapsed alveoli with thick wall **(A)** and few dilated ones with destroyed wall **(D) (C)** An image shows bronchioles (Br) lined by hyperplastic epithelium. The lumen containing cellular epithelial debris and inflammatory cells aggregate (crossed arrow) **(D)** An image shows markedly congested pulmonary blood vessels (BV) with marked perivascular cuffing by mononuclear inflammatory cellular infiltrate, increased wall thickness (arrow).

Figure 4 shows photomicrograph of H&E stained lung from leflunomide 10 mg/kg group. Panel (A) shows markedly distorted lung architecture with heavy mononuclear inflammatory cell infiltrate. Panel (B) shows multiple areas of diffuse alveolar damage, thickened pulmonary interstitium with inflammatory cells and interstitial alveolar hemorrhage. Panel **(C)** shows bronchioles are lined by hyperplastic epithelium. The lumen is shown containing cellular epithelial debris and inflammatory cells aggregate. Panel **(D)** An image shows markedly congested pulmonary blood vessels with marked perivascular cuffing by mononuclear inflammatory cellular infiltrate, increased wall thickness.

**FIGURE 5:**
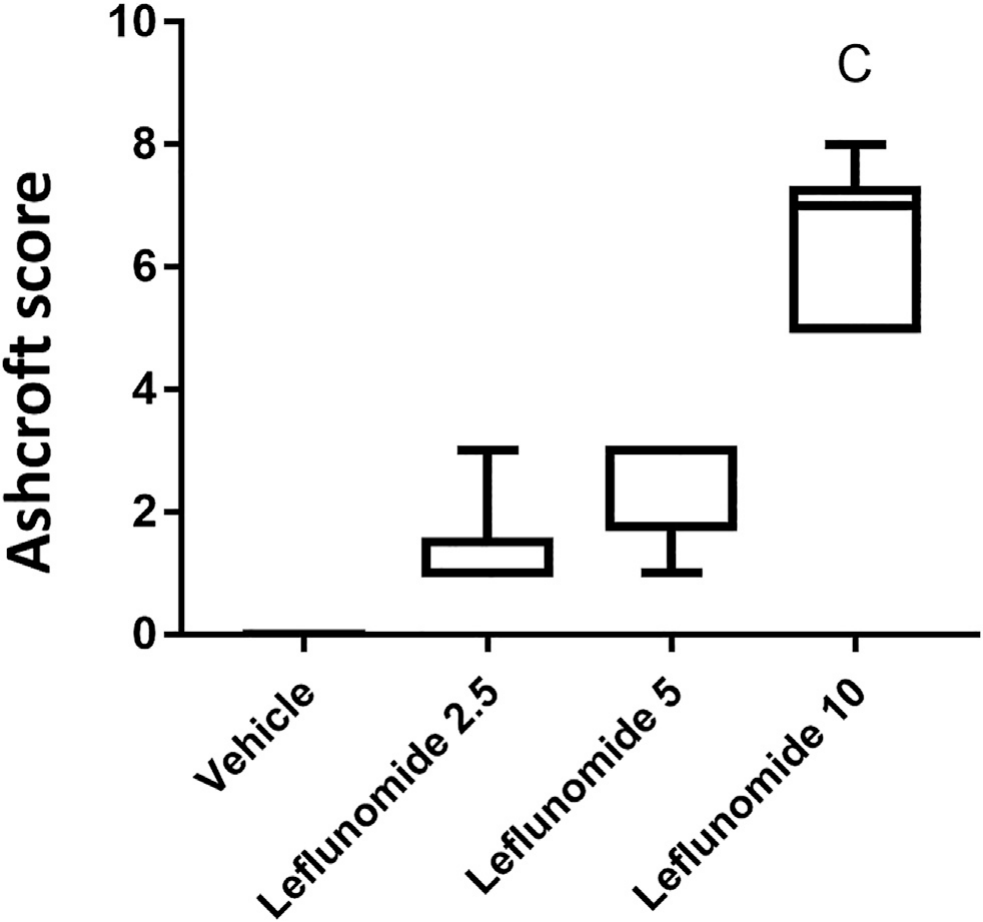
The Ashcroft scores for the H&E stained lung sections**.** Data are box-plots representing the medians and quartiles. Statistical analysis was done with Kruskal-Wallis ANOVA and Dunn’s test. a: vs. vehicle group at *p* < 0.05.

Figure 5 shows based on Ashcroft scoring system revealed that the vehicle control group 1) in which mice received CMC solution for 8 weeks, 100% of the mice showed normal lung tissue with no fibrosis or inflammatory cellular infiltrate. In mice gavaged with leflunomide (2.5 mg/kg), lung tissues from 5 mice (83.33%) revealed grade 1 fibrosis and 1 case (16.66%) showed grade 3 fibrosis. In mice received leflunomide (5 mg/kg), lung tissues of 4 (66.66%) mice lung tissues showed grade 5 fibrosis, 1 mouse (16.66%) lung tissues showed grade 3 fibrosis and 1 mouse (16.6%) lung tissues exhibited grade 1 fibrosis. In mice received leflunomide (10 mg/kg) every other day. Lung tissues from 2 mice (33.33%) showed grade 5 fibrosis, lungs from 5 mice (83.33%) revealed grade 7 fibrosis and only one mouse (16.66%) lung tissue showed complete obliteration of the lung tissue by fibrosis (grade 8).

**FIGURE 6:**
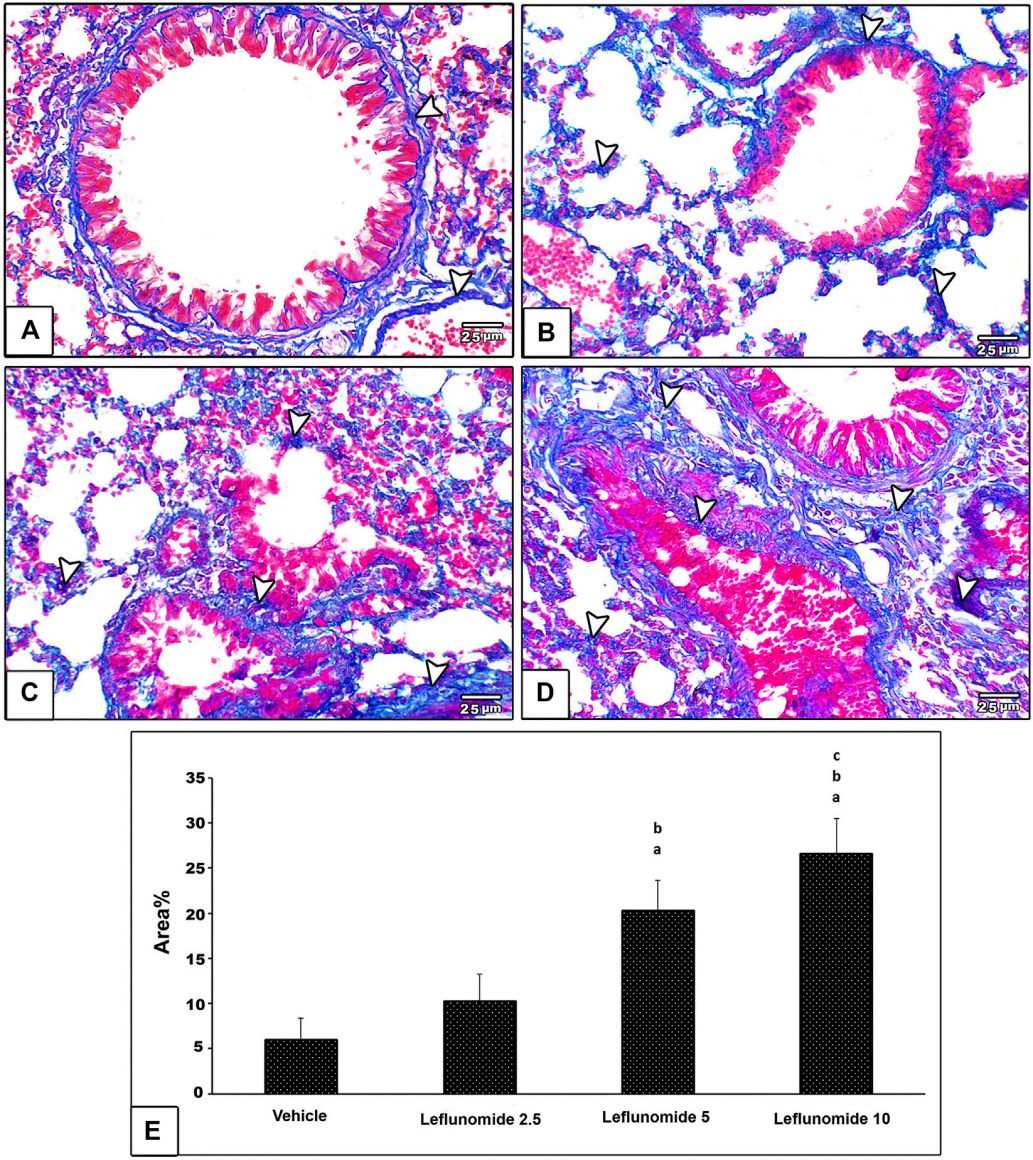
Photomicrograph of Masson’s trichrome stained lung sections **(A)** An image from the vehicle control group shows minimal amount of collagen fibers (blue color) (arrow head) in pulmonary interstitium surrounding blood vessels and bronchioles **(B)** An image from group ii shows mild amount of fibrosis in pulmonary interstitium, perivascular and peribronchiolar areas **(C)** An image from group iii shows moderate increased collagen deposition **(D)** An image from group iv shows marked deposition of collagen fibers in lung section **(E)** A column chart representing the mean area ± SDM, **(A)**: vs. the vehicle group, **(B)**: vs. the leflunomide 2.5 mg per kg group, **(C)**: vs. the leflunomide 5 mg per kg group at *p* < 0.05.

Masson’s trichrome staining indicated minimal amount of collagen fibers in the vehicle control group; collagen was found in pulmonary interstitium surrounding blood vessels and bronchioles (Figure 6A). Leflunomide (2.5 mg/kg) group displayed mild fibrosis in pulmonary interstitium, perivascular and peribronchiolar areas (Figure 6B). Mice gavaged with leflunomide (5 mg/kg) displayed moderately increased collagen deposition (Gig 6C). Leflunomide (10 mg/kg) group exhibited marked deposition of collagen fibers in lung section. Statistical analysis highlighted significant increments in the area of collagen presence in the leflunomide (5 and 10 mg/kg) groups. Importantly the level of collagen deposition in leflunomide (10 mg/kg) group was significantly higher than that observed in leflunomide (5 mg/kg) group (Figure 6E).

**FIGURE 7:**
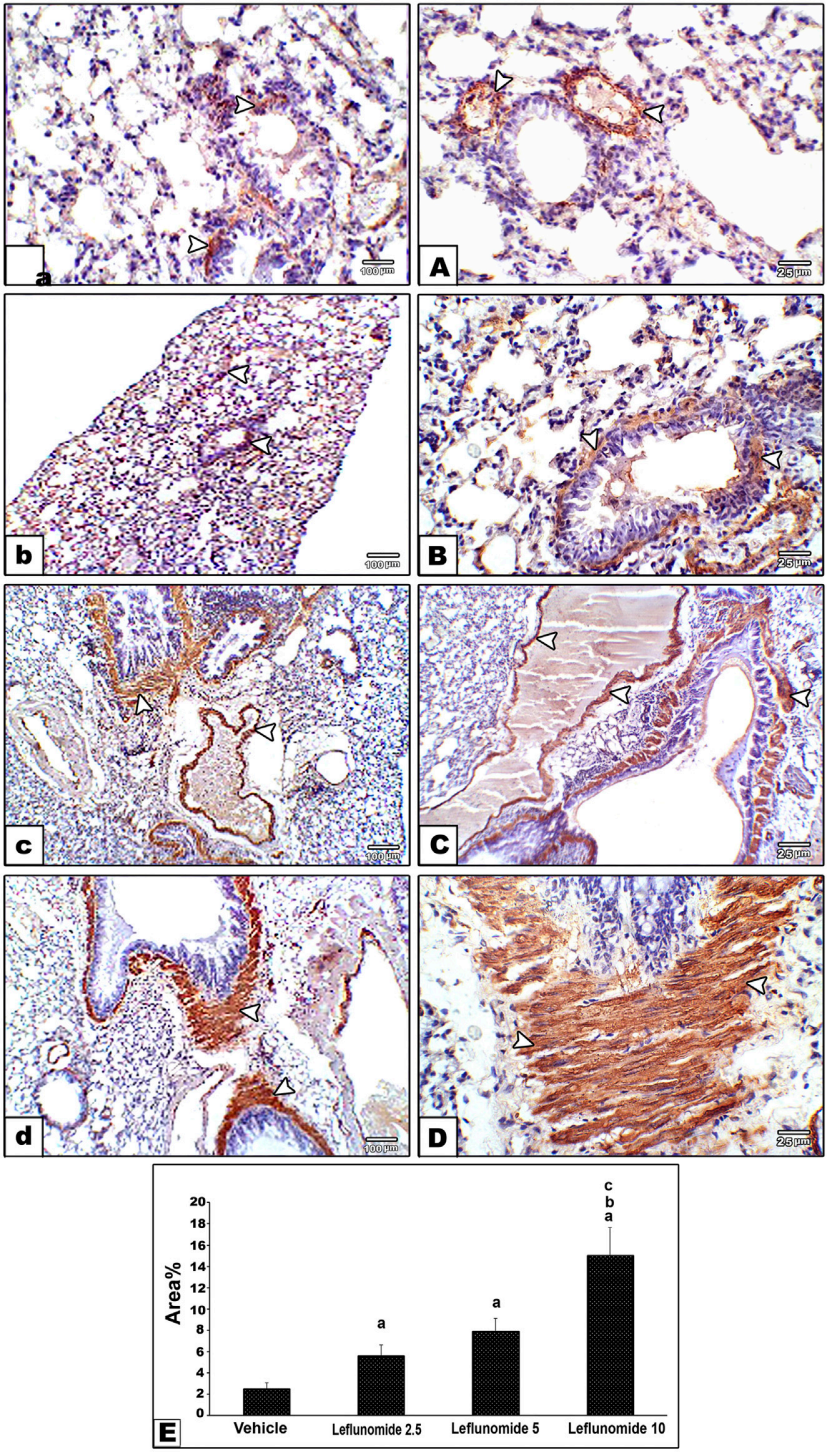
Immunohistochemical staining for α-smooth muscle actin in lung sections **(A)** An image from the vehicle control group (group I) shows mild cytoplasmic expression of smooth muscle actin antibody in perivascular and peribronchiolar muscle coat with also expression in scattered few mesenchymal cells in interalveolar septa. Compared to its gradually and progressively increased expression in leflunomide 2.5 group **(B)**, leflunomide 5 group **(C)** and leflunomide 10 group **(D) (E)** A column chart representing the mean area ± SDM, **(A)**: vs. the vehicle group, **(B)**: vs. the leflunomide 2.5 mg per kg group, **(C)**: vs. the leflunomide 5 mg per kg group at *p* < 0.05.

Immunohistochemical staining for α-SMA in lung specimens showed mild cytoplasmic expression in the vehicle control group that was found in the perivascular and peribronchiolar muscle coat and found in scattered few mesenchymal cells in interalveolar septa (Figure 7A). Leflunomide (2.5, 5 and 10 mg/kg) groups showed progressive increases in the lung immunostaining for α-SMA (Figures 7B–D ). Statistical analysis for the area of α-SMA immunostaining indicated a significant increment in the leflunomide (10 mg/kg) group compared to the vehicle group (Figure 7E).

**FIGURE 8:**
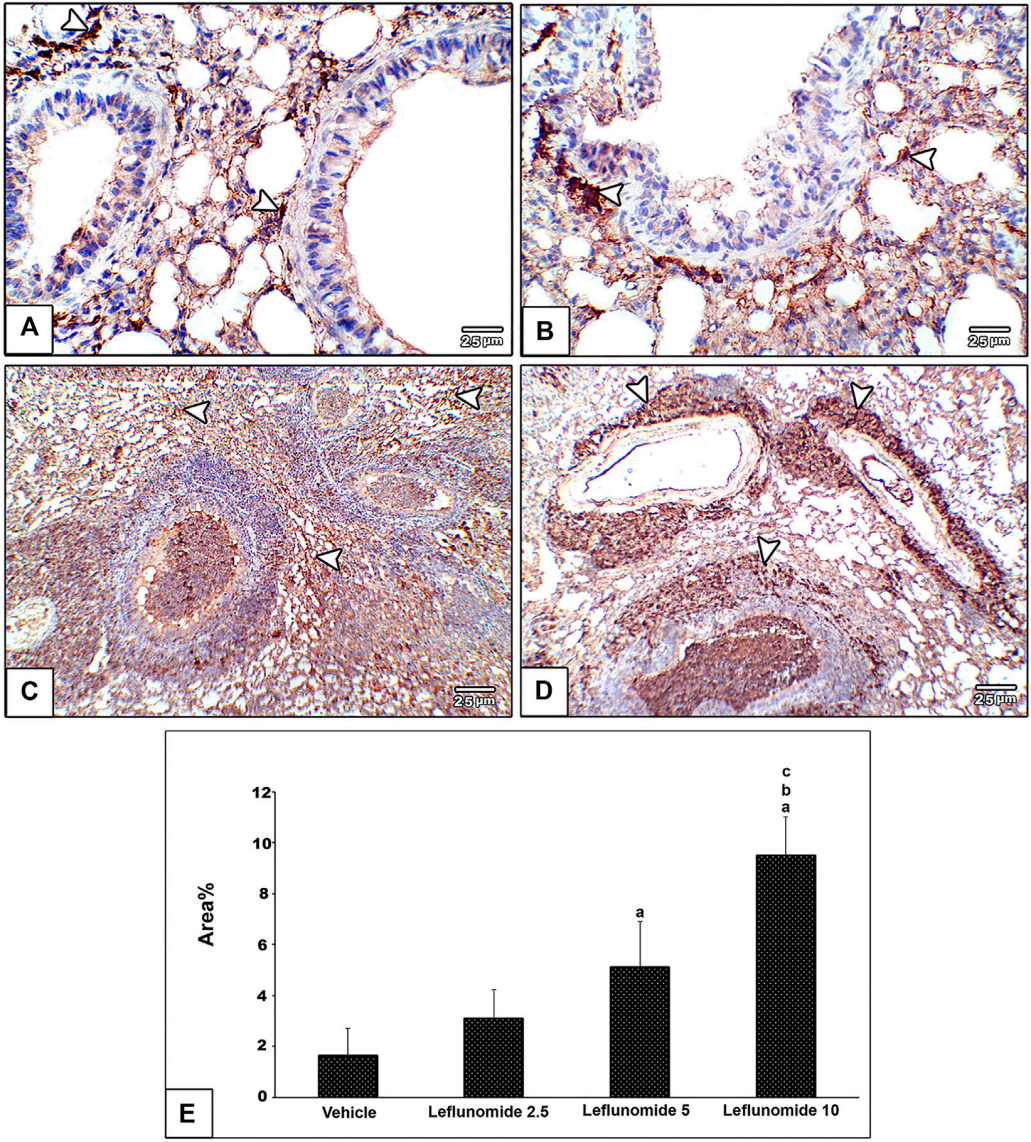
Immunohistochemical staining of vimentin in lung sections **(A)** An image from the vehicle group shows weak expression of vimentin compared to moderately increased expression of vimentin in mesenchymal cells in inter alveolar septa, perivascular and peribronchiolar spaces in leflunomide 2.5 group **(B)**, leflunomide 5 group **(C)** and markedly increased in leflunomide 10 group **(D) (E)** A column chart representing the mean area ± SDM, **(A)**: vs. vehicle group, **(B)**: vs. the leflunomide 2.5 mg per kg group, **(C)**: vs. the leflunomide 5 mg per kg group at *p* < 0.05.

Immunohistochemical staining of vimentin in lung sections revealed weak expression of vimentin in the vehicle group (Figure 8A). Moderate formation of vimentin was observed in leflunomide (2.5 and 5 mg/kg) group in mesenchymal cells in inter alveolar septa, perivascular and peribronchiolar spaces in groups (Figures 8B,C). A marked increase in vimentin expression was observed in leflunomide (10 mg/kg) group (Figure 8D). The vimentin stained areas measured in leflunomide (5 and 10 mg/kg) groups were significantly greater than that measured in the vehicle group (Figure 8E).

**FIGURE 9:**
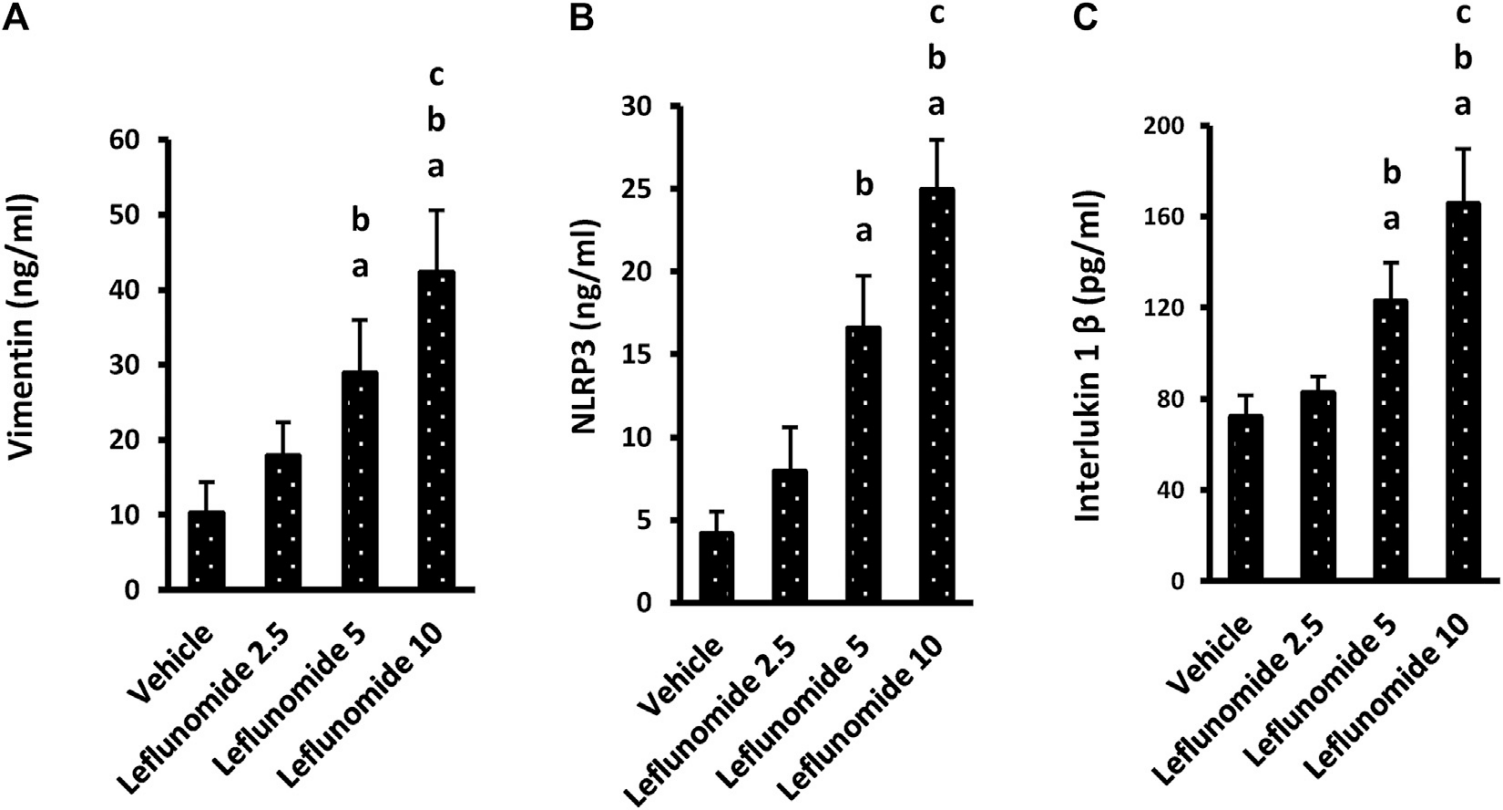
The level of the inflammatory markers in lung homogenates. **(A)** vimentin, **(B)** NLRP3 and **(C)** interlukin-1β. Column chart representing the mean area ± SDM, **(A)**: vs. the vehicle group, **(B)**: vs. the leflunomide 2.5 mg per kg group, **(C)**: vs. the leflunomide 5 mg per kg group at *p* < 0.05.

One-way ANOVA highlighted a difference among the study groups [F (3,20) = 29.22, F (3,20) = 53.11, F (3,20) = 41.81] in lung vimentin, NLRP3 and IL-1β. ELISA assays indicated significant increases in these three markers in mice treated with 5 or 10 mg/kg of leflunomide (Figures 9A–C). Importantly, the higher dose of leflunomide (10 mg/kg).

**FIGURE 10:**
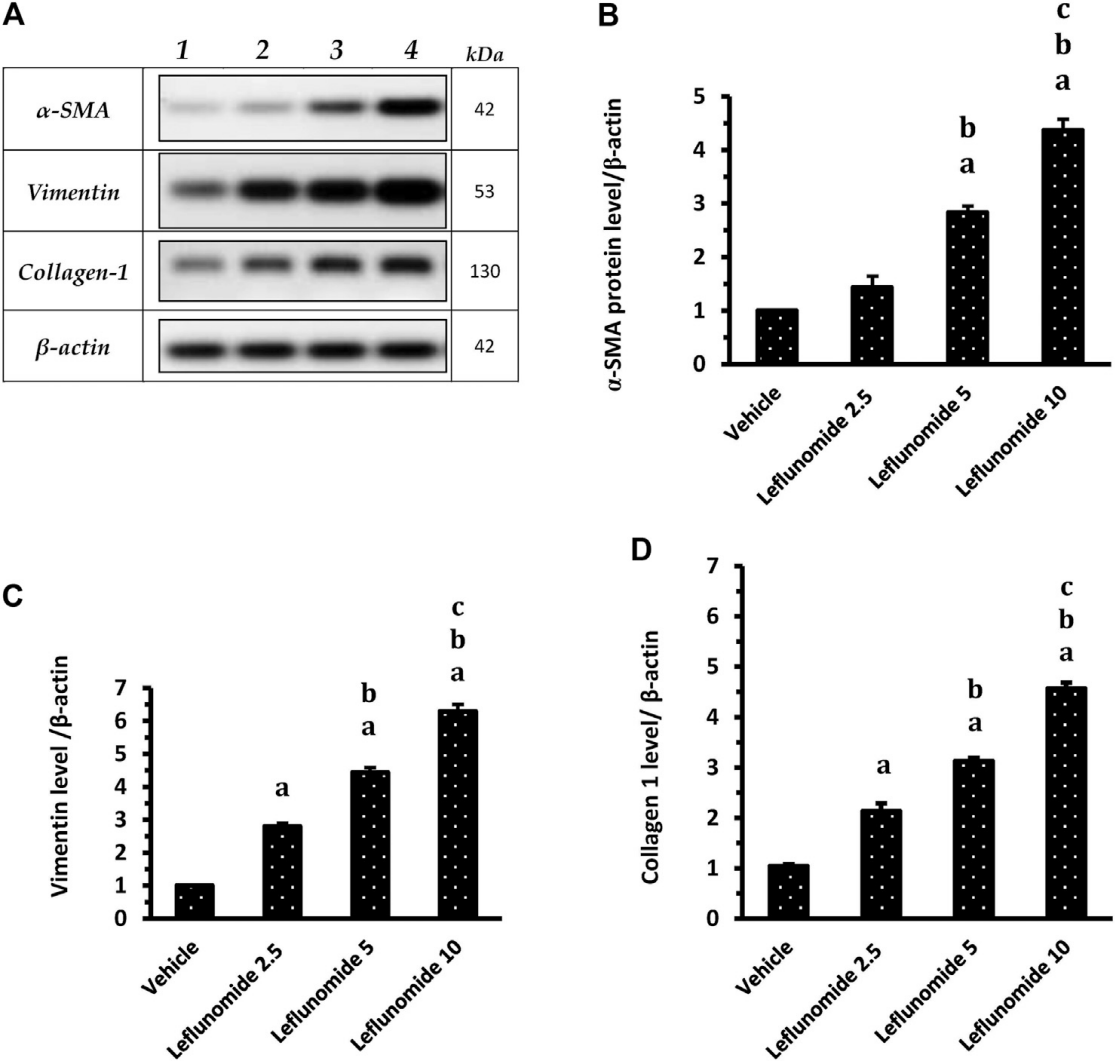
Western blot analysis for α-SMA, vimentin and collagen 1 proteins. **(A)**: Western blot gels of the target genes in (1) vehicle group, (2) leflunomide 2.5 mg/kg group, (3) leflunomide 5 mg/kg group and (4) leflunomide 10 mg/kg group. (**B–D)**: Column charts , vimentin and collagen 1 proteins relative to β-actin. The quantification data are presented as mean ± SDM. **(A)**: vs. the vehicle group, **(B)**: vs. leflunomide 2.5 mg per kg group, **(C)**: vs. leflunomide 5 mg per kg group at *p* < 0.05. α-SMA: α-smooth muscle actin.

Figure 10A demonstrates Western blot gels for the target proteins (α-SMA, vimentin and collagen 1) in the lung specimens compared to the house keeping gene. The column charts demonstrate greater band density for the target proteins in the different groups SMA level in leflunomide 5 and 10 mg/kg groups was greater than that observed in the vehicle group and the leflunomide 2.5 mg/kg group (Figure 10B ). For vimentin and collagen-1 levels, leflunomide produced dose-dependent increases in their levels compared to the vehicle groups (Figures 10C,D).

## Discussion

The currents study highlighted for the first time the lung injury and fibrosis in healthy mice administered leflunomide. This was proved by histopathologic manifestations recorded in H&E stained lung sections and gradual and then significant rise in Ashcroft score. In addition, lung specimens showed dose-dependent increases in fibrotic changes as seen in Masson’s trichrome staining and α-SMA immunostaining. Furthermore, the study aimed to explore some mechanisms behind this toxic effect and found that vimentin induced NLRP3 plays a role in such toxicity and led to increased pulmonary IL-β.

Regarding leflunomide pulmonary toxicity, two systematic reviews published different results. One systematic review established a relationship between the presence or deteriorating ILDs and leflunomide. Bilateral ground-glass opacities (GGO) plus diffused destructed alveoli were apparent in radiologic and histopathologic investigations due to the use of leflunomide (Raj and Nugent, 2013). Other studies declared that methotrexate, leflunomide and biologic DMARDs carry risks in rheumatoid arthritis-induced interstitial lung diseases (England and Hershberger, 2020) and leflunomide use was accompanied with fast onset hypersensitivity pneumonia and new-onset or progression of pre-existing ILDs (Cassone et al., 2020). On the contrary Conway et al. declared no relationship between leflunomide use and amplified hazard in eight different controlled trials; they mentioned that leflunomide did not augment the total or infectious respiratory adverse effects (Conway et al., 2016).

Our study demonstrated that use of leflunomide for 8°weeks in has induced histopathologically evident lung injury in the form of distorted architecture, marked inflammatory cells infiltrate and increase collagen content in the lung tissues especially in groups given high doses of leflunomide; this was in agreement with some previous reports (Raj and Nugent, 2013; Cassone et al., 2020; England and Hershberger, 2020). In order to investigate the possible mechanism of leflunomide induced lung injury we measured vimentin, NLRP3 and IL-1β in lung homogenate and their levels were found to be significantly elevated with large doses of leflunomide given to healthy mice every other day for 8°weeks.

Activation of the NLRP3 inflammasome requires TLRs signaling to activate NF-kB and upregulate NLRP3 and pro-IL-1β (Bauernfeind et al., 2011). Indeed, activation of the NLRP3 inflammasome and following IL-1β maturation is implicated in ALI, resulting in inflammation and fibrosis (dos Santos et al., 2015). This is characterized by inflammation, IL-1β release, alveolar epithelial cell injury, hyperplasia of alveolar type 2 cells, accumulation of fibroblasts, deposition of collagen and scar formation. IL-1β and IL-18 are related cytokines that produce many biological effects related to inflammation and autoimmune processes. IL-1β contributes to the generation of both the systemic and local reactions toward infection or injury through the generation of fever, activating lymphocytes and promotion of leukocytic infiltration (Wiese et al., 2017).

Similarly, activation of the NLRP3 inflammasome takes place as a reaction toward a broad spectrum of infectious organisms known to cause ALI. These infectious organisms include bacterial pathogens like *Pseudomonas aeruginosa Staphylococcus aureus* and *Mycobacterium tuberculosis* (Miller et al., 2007; Mishra et al., 2010; Cohen and Prince, 2013), the influenza A virus (Allen et al., 2009) and fiber associated lung inflammation (Sayan and Mossman, 2015).

It was shown that vimentin is essential for activating the NLRP3 inflammasome. A direct protein–protein interactions between NLRP3 and vimentin was documented; this provides new insights toward pulmonary inflammation and/or fibrosis (dos Santos et al., 2015).

The wide variety of pathogens which are recognized as activators for the NLRP3 inflammasome proposes that NLRP3 senses microbes by an indirect way through searching for danger-associated molecular forms that are produced/released from the host subsequent to cell or tissue damage (Lamkanfi and Dixit, 2009a; Lamkanfi and Dixit, 2009b). For instance, uric acid release from cells subjected to bleomycin generates the NLRP3 inflammasome, resulting in IL-1β maturation and inflammation (Gasse et al., 2009). Bleomycin administration to human patients and animals leads to L-1β release from damaged cells leading to fabrication of transforming growth factor β which is known as a pro-fibrotic cytokine and has a role in developing pulmonary fibrosis (Wynn, 2011). Furthermore, exposure to crystalline molecules like asbestos (Dostert et al., 2008) and silica (Cassel et al., 2008) results in pulmonary fibrosis (Liu et al., 2013) that is regulated by the NLRP3 inflammasome (Cassel et al., 2008; Dostert et al., 2008).

In previous study inspected the action of vimentin in ALI using different models in which NLRP3 inflammasome activation is required. They demonstrated that key pathophysiological circumstances in ALI like inflammatory reactions, IL-1β production, epithelial barrier permeability, remodeling and fibrosis are diminished in the lungs of Vim−/−mice when they were confronted with bleomycin, lipopolysaccharide or subjected to asbestosis (dos Santos et al., 2015). In agreement, bone marrow chimeric mice missing vimentin, exposed to bleomycin showed lessened IL-1β level and mitigated lung injury and fibrosis. Further, an *in vitro* study reported declined levels of active caspase-1 and IL-1β were detected in Vim−/− and vimentin-knockdown macrophages (dos Santos et al., 2015). Moreover, another study concluded that vimentin arbitrates NLRP3 activation and encourages brain inflammatory reactions and neuron injury in mice suffering from CNS EV71 infection. In comparison to the control mice, the VIM−/− EV71-infected mice had non-significant alterations in the expression of NLRP3, caspase-1 or even IL-1β. Differently, the wild-type EV71-infected mice exhibited noticeably amplified central expression of NLRP3, IL-1β and caspase-1. Furthermore, there was milder neuronal damage in infected VIM−/− mice when compared to the wild-type mice (Xiao et al., 2018).

One of the strength points for our study is that it is limited to evaluating the dose-related effect of leflunomide in healthy mice, limiting as much as possible the presence of other confounding factors or factors that could modify the observed effects. In conclusion, the present study highlighted the dose-dependent lung injury prompted by leflunomide in mice and provided an evidence for such toxicity that may be of clinical importance if appropriate clinical data will be available. Until this time, the current study suggests that rheumatic patients on leflunomide therapy should monitor their pulmonary function and consult their doctors if they progressively suffer from any respiratory symptoms. In addition, the findings of the present study highlighted a novel role for vimentin-induced NLRP3 inflammasome in the lung injury produced by leflunomide in mice. This point opens avenues for new therapeutic tools that may retard drug-induced ALI by inhibiting this pathway.

Further studies are warranted to confirm the current findings by concurrent administration of an NLRP3 antagonist or siRNA and look whether they may alleviate pulmonary inflammation induced by leflunomide.

## Data Availability

The raw data supporting the conclusions of this article will be made available by the authors, without undue reservation.
