# Supplementary material for: Leflunomide Induces Dose-Dependent Lung Injury in Mice via Stimulating Vimentin and NLRP3 Inflammasome Production
Source: Front Pharmacol. 2021 Apr 23;12:631216. doi: 10.3389/fphar.2021.631216 (PMC8115235; doi:10.3389/fphar.2021.631216)
Supplement: Supplementary file 1 [file image3.jpeg]

Frontiers | Leflunomide induces dose-dependent lung injury in mice via stimulating vimentin and NLRP3 inflammasome production | Pharmacology


Login
 / 
Register

- About
- Journals
- Research Topics
- Articles
- More

Submit

Login
 / 
Register

Submit

**Impact Factor 4.225** | **CiteScore 5.0**More on impact ›

|  |  |
| --- | --- |
| Frontiers in Pharmacology | Respiratory Pharmacology |

Toggle navigation


Section


- (current)Section
- About
- Articles
- Research topics
- For authors 
  - Why submit?
  - Fees
  - Article types
  - Author guidelines
  - Review guidelines
  - Submission checklist
  - Contact editorial office
  - Submit your manuscript
- Editorial board

- *Article alerts*

Articles


**Suggest a Research Topic >**

- 105
  total views

 View Article Impact

**Suggest a Research Topic >**

##### SHARE ON

- Facebook

  0
- Twitter

  0
- LinkedIn

  0
- AddThis

  New


## Original Research ARTICLE

Front. Pharmacol.
| doi: 10.3389/fphar.2021.631216

# Leflunomide induces dose-dependent lung injury in mice via stimulating vimentin and NLRP3 inflammasome production Provisionally accepted The final, formatted version of the article will be published soon. **Notify me**

Mohamed El-Sherbiny1, 2,  Hoda Atef3,  Mohamed Ahmed Eladl4,  Abdelaty S. Mohamed1, 5,  Mohamed El-Shafey2, 6,  Howaida S. Ali7, 8,  Sawsan A. Zaitone8, 9\*,  Suliman Y. Alomar10\*, Saeed A. Alqahtani11, 
 Sheka Y. Aloyouni12 and  Mohammed A. Attia13, 14

- 1Department of Basic Medical Sciences, Faculty of Medicine, Almaarefa University, Saudi Arabia
- 2Department of Anatomy and Embryology, Faculty of Medicine, Mansoura University, Egypt
- 3Department of Histology, Faculty of Medicine, Mansoura University, Egypt
- 4Department of Basic Medical Sciences, College of Medicine, University of Sharjah, United Arab Emirates
- 5Department of Pathology, Faculty of Medicine, Mansoura University, Egypt
- 6Department of Physiological Sciences, Fakeeh College for Medical Sciences, Saudi Arabia
- 7Department of Pharmacology, Faculty of Pharmacy, Assiut University, Egypt
- 8Department of Pharmacology and Toxicology, Faculty of Pharmacy, University of Tabuk, Saudi Arabia
- 9Department of Pharmacology and Toxicology, Suez Canal University, Egypt
- 10Department of Zoology, College of Science, King Saud University, Saudi Arabia
- 11Department of Physiology, Faculty of Medicine, Taibah University, Saudi Arabia
- 12Health Science Research Center, Princess Nourah bint Abdulrahman University, Saudi Arabia
- 13Department of Pharmacology, Faculty of Medicine, Mansoura University, Egypt
- 14Department of Pharmacology, College of Medicine, Almaarefa University, Saudi Arabia

Recently, the therapeutic importance of the anti-rheumatic drug, leflunomide, has been increased after the involvement of leflunomide in treating other autoimmune diseases and its promising role in retarding human malignancies. Few studies have focused on the safety in human or animals without clear outlining of the pathologic features on target organs. One clinical study related leflunomide with significant pulmonary complications in predisposed individuals. The current study examined the dose-dependent lung injury produced by leflunomide in healthy mice. Albino mice were allocated into four different groups. Group (1): Vehicle control group, Group (2-4): mice received leflunomide (2.5, 5 or 10 mg/kg), respectively, for 8 weeks and then lungs were dissected from the mice for histopathological examination and fibrosis evaluation (Masson’s trichrome staining and α-smooth muscle actin immunohistochemistry). Enzyme linked immunosorbent assay was used to assess the vimentin and other inflammatory factors in the lung homogenate whereas Western blot analysis was employed to assess α-smooth muscle actin, vimentin and collagen 1. Results indicated that leflunomide induced dose-dependent pulmonary injury and the high dose and increased the vimentin, inflammatory markers (NLRP3 and interlukin-1β). Histologic examination showed distorted architecture, marked inflammatory cells infiltrate and increase collagen content. The findings were supported by Western blotting and the immunohistochemical study which showed greater pulmonary α-smooth muscle actin and vimentin content. In conclusion, the current results highlighted that leflunomide produced dose-dependent pulmonary toxicities that requires further investigation of the nature of injury.

Keywords: 
Collagen 1, Fibrosis, Leflunomide, Lung Injury, Mouse, NLRP3, Vimentin

Received: 20 Nov 2020;
Accepted: 10 Mar 2021.

Copyright: © 2021 El-Sherbiny, Atef, Eladl, Mohamed, El-Shafey, Ali, Zaitone, Alomar, Alqahtani, Aloyouni and Attia. This is an open-access article distributed under the terms of the Creative Commons Attribution License (CC BY). The use, distribution or reproduction in other forums is permitted, provided the original author(s) and the copyright owner(s) are credited and that the original publication in this journal is cited, in accordance with accepted academic practice. No use, distribution or reproduction is permitted which does not comply with these terms.

\* Correspondence: 
  
 Prof. Sawsan A. Zaitone, Suez Canal University, Department of Pharmacology and Toxicology, Ismaïlia, Egypt, Sawsan\_zaytoon@pharm.suez.edu.eg   
 Dr. Suliman Y. Alomar, Department of Zoology, College of Science, King Saud University, Riyadh, 11451, Saudi Arabia, syalomar@ksu.edu.sa

1 Comments

User

- Comment deleted on 1:37 AM, 11 March 2021

Write a comment...

Add

##### COMMENTARY

##### ORIGINAL ARTICLE

##### People also looked at

## Evening Primrose Oil Ameliorates Hyperleptinemia and Reproductive Hormone Disturbances in Obese Female Rats: Impact on Estrus Cyclicity

Hebatallah H. Atteia, Sharifa Alzahrani, Nagla A. El-Sherbeeny, Amal M. Youssef, Noha E. Farag, Eman T. Mehanna, Reda Elhawary, Gehan A. Ibrahim, Amr Elmistekawy and Sawsan A. Zaitone

## Relationship Between Smoking Habit and Sperm Parameters Among Patients Attending an Infertility Clinic

Rehana Rehman, Nida Zahid, Sofia Amjad, Mukhtiar Baig and Zohair Jamil Gazzaz

## Supplement With Calcium or Alendronate Suppresses Osteopenia Due to Long Term Rabeprazole Treatment in Female Mice: Influence on Bone TRAP and Osteopontin Levels

Aly A.M. Shaalan, Mohamed El-Sherbiny, Taghrid B. El-Abaseri, Mohamed Z. Shoaeir, Tarek M. Abdel-Aziz, Magda I. Mohamed, Sawsan A. Zaitone and Hala M. F. Mohammad

## Midgut Epithelial Dynamics Are Central to Mosquitoes’ Physiology and Fitness, and to the Transmission of Vector-Borne Disease

Bretta Hixson, Mabel Laline Taracena and Nicolas Buchon

## Optimization of Inulin Hydrolysis by Penicillium lanosocoeruleum Inulinases and Efficient Conversion Into Polyhydroxyalkanoates

Iolanda Corrado, Nicoletta Cascelli, Georgia Ntasi, Leila Birolo, Giovanni Sannia and Cinzia Pezzella

**Suggest a Research Topic >**

×

#### Supplementary Material

  

There is no supplementary material currently available for this article

Loading supplemental data...

  

|  | File Name |  |
| --- | --- | --- |
|  | Image 1.JPEG |  |
|  | Image 2.JPEG |  |
|  | Image 3.JPEG |  |
|  | Image 4.JPEG |  |

  

Close

- About Frontiers
- Institutional Membership
- Books
- News
- Frontiers' social media
- Contact
- Careers
- Submit
- Newsletter
- Help Center
- Terms & Conditions
- Privacy Policy

© 2007 - 2021 Frontiers Media S.A. All Rights Reserved

### Privacy Preference Center

Our website uses cookies that are necessary for its operation. Additional cookies are only used with your consent. These cookies are used to store and access information such as the characteristics of your device as well as certain personal data (IP address, navigation usage, geolocation data) and we process them to analyse the traffic on our website in order to provide you a better user experience, evaluate the efficiency of our communications and to personalise content to your interests. Some cookies are placed by third-party companies with which we work to deliver relevant ads on social media and the internet. Click on the different categories' headings to change your cookie preferences. Click on "More Information" if you wish to learn more about how data is collected and shared.
More information

### Manage Consent Preferences

#### Strictly Necessary Cookies

Always Active

These cookies are necessary for the website to function and cannot be switched off in our systems. They are usually only set in response to actions made by you which amount to a request for services, such as setting your privacy preferences, logging in or filling in forms. You can set your browser to block or alert you about these cookies, but some parts of the site will not then work. These cookies do not store any personally identifiable information.

#### Analytics Cookies

Analytics Cookies

These cookies allow us to count visits and traffic sources so we can measure and improve the performance of our site. They help us analyse which pages are the most and least popular and see how visitors move around the site.    All information these cookies collect is aggregated and therefore anonymous.

#### Functional Cookies

Functional Cookies

These cookies enable the website to provide enhanced functionality and personalisation. They may be set by us or by third party providers whose services we have added to our pages. If you do not allow these cookies then some or all of these services may not function properly.

#### Advertising Cookies

Advertising Cookies

These cookies may be set through our site by our advertising partners. They may be used by those companies to build a profile of your interests and show you relevant adverts on other sites.    They do not store directly personal information, but are based on uniquely identifying your browser and internet device. If you do not allow these cookies, you will experience less targeted advertising.

### Back Button Performance Cookies

Vendor Search  Search Icon

Filter Icon

Clear

checkbox label label

Apply Cancel

Consent Leg.Interest

checkbox label label

checkbox label label

checkbox label label

Confirm My Choices
