# Supplementary figures and images for: Leflunomide Induces Dose-Dependent Lung Injury in Mice via Stimulating Vimentin and NLRP3 Inflammasome Production
Source: Front Pharmacol. 2021 Apr 23;12:631216. doi: 10.3389/fphar.2021.631216 (PMC8115235; doi:10.3389/fphar.2021.631216)

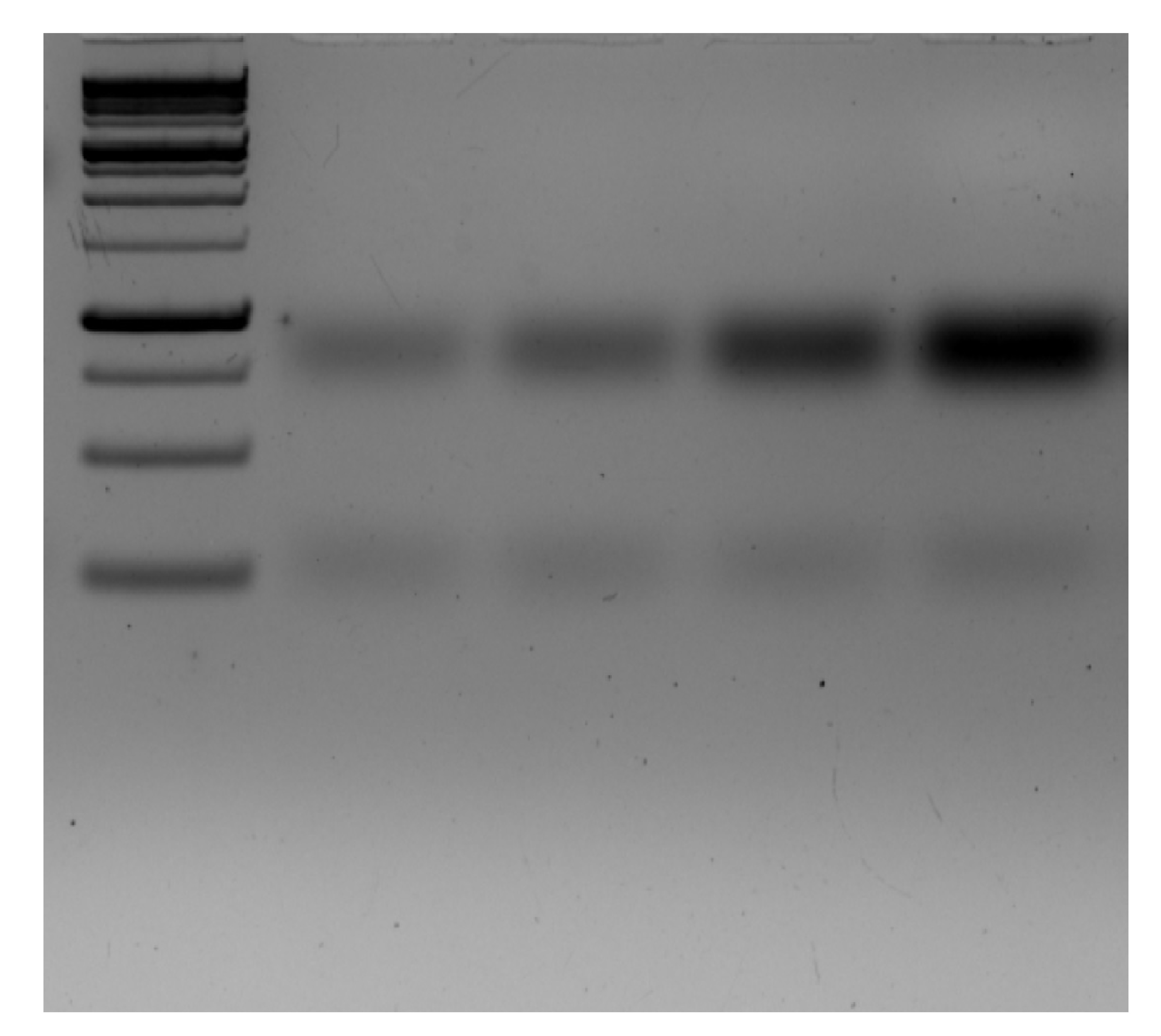

Supplement: Supplementary file 2 [file image1.jpeg]
